# Plant Defenses and Predation Risk Differentially Shape Patterns of Consumption, Growth, and Digestive Efficiency in a Guild of Leaf-Chewing Insects

**DOI:** 10.1371/journal.pone.0093714

**Published:** 2014-04-09

**Authors:** Ian Kaplan, Scott H. McArt, Jennifer S. Thaler

**Affiliations:** 1 Department of Entomology, Purdue University, West Lafayette, Indiana, United States of America; 2 Departments of Entomology and Ecology & Evolutionary Biology, Cornell University, Ithaca, New York, United States of America; French National Institute for Agricultural Research (INRA), France

## Abstract

Herbivores are squeezed between the two omnipresent threats of variable food quality and natural enemy attack, but these two factors are not independent of one another. The mechanisms by which organisms navigate the dual challenges of foraging while avoiding predation are poorly understood. We tested the effects of plant defense and predation risk on herbivory in an assemblage of leaf-chewing insects on *Solanum lycopersicum* (tomato) that included two Solanaceae specialists (*Manduca sexta* and *Leptinotarsa decemlineata*) and one generalist (*Trichoplusia ni*). Defenses were altered using genetic manipulations of the jasmonate phytohormonal cascade, whereas predation risk was assessed by exposing herbivores to cues from the predaceous stink bug, *Podisus maculiventris*. Predation risk reduced herbivore food intake by an average of 29% relative to predator-free controls. Interestingly, this predator-mediated impact on foraging behavior largely attenuated when quantified in terms of individual growth rate. Only one of the three species experienced lower body weight under predation risk and the magnitude of this effect was small (17% reduction) compared with effects on foraging behavior. *Manduca sexta* larvae, compensated for their predator-induced reduction in food intake by more effectively converting leaf tissue to body mass. They also had higher whole-body lipid content when exposed to predators, suggesting that individuals convert energy to storage forms to draw upon when risk subsides. In accordance with expectations based on insect diet breadth, plant defenses tended to have a stronger impact on consumption and growth in the generalist than the two specialists. These data both confirm the ecological significance of predators in the foraging behavior of herbivorous prey and demonstrate how sophisticated compensatory mechanisms allow foragers to partially offset the detrimental effects of reduced food intake. The fact that these mechanisms operated across a wide range of plant resistance phenotypes suggests that compensation is not always constrained by reduced food quality.

## Introduction

Although stress is a near-omnipresent feature of life in the natural world, the type of stressor and its impact on organismal ecology, behavior, and physiology can be quite variable. Quantifying this variation is complicated by the fact that if the impacts of simultaneous or sequential exposure to multiple stressors are non-additive, their outcome cannot be accurately predicted from testing each factor in isolation. For example, predator-induced stress exacerbates the effect of anthropogenic pollutants (e.g., pesticides) on development of amphibians and invertebrates [1]–[4]. A recent meta-analytical synthesis of 112 factorial design experiments assessing animal mortality in response to multiple stressors revealed that non-additivity was the rule rather than the exception with synergism or antagonism reported in >75% of all cases [5]. Because animals are routinely exposed to a large number of sublethal stress agents in nature, the challenge is to identify the ecologically relevant ones and uncover their singular and combined impacts.

For herbivores, the two biotic variables most strongly linked to individual fitness are food resources and natural enemies. Accordingly, these bottom-up and top-down factors have become primary themes in research exploring the ecology and evolution of herbivory [6], [7]. Put simply, the success of plant-feeders reflects their ability to acquire sufficient amounts of high quality food while avoiding becoming another’s meal in the process. While the consequences of plant variation have long been recognized [8]–[11], the impact of chronic predation risk and the interactions between predator- and food-induced stress are poorly understood [12], [13]. It is now apparent, however, that predation risk can have far-reaching impacts on growth, survival and/or reproduction [14]–[18]. These impacts are often attributed to altered prey foraging: increasingly risk-averse behavior in the presence of predators can lead to reduced food intake [19], [20]. Predators also elicit more subtle physiological responses in threatened prey [21], [22]; these include, elevated metabolic rate [23] and oxidative stress [24], [25], both of which may amplify energetic costs of engaging in anti-predator activities.

The manner by which plant quality interactively shapes responses to predation risk is unclear, despite the fact that herbivores are habitually faced with such integrative decision making [26]–[29]. Part of the difficulty in teasing apart this relationship is that responses to variable plant quality alone are notoriously challenging to predict. Low quality plants can provoke compensatory feeding with correspondingly greater tissue damage compared with high quality plants [30]–[32]; however, the nature of this response depends on the specific plant traits at play. The mixture of toxins, digestibility-reducing compounds, free nitrogen, and structural defenses largely determines plant quality, and the relative importance of these traits ultimately drives herbivore feeding behavior [33]. Another problem with experimentally dissecting resource-risk interactions is that the two stressors are not independent, making it difficult to evaluate their combined impact. The fact that the same phytohormonal pathway mediating carnivore attraction to herbivore-damaged plants also reduces leaf nutritive content [34], for instance, suggests that low food quality may sometimes be functionally linked with heightened predation risk. For communities possessing mobile foragers, the opposite scenario may be the case. Grasshoppers seek enemy-free space on lower quality herbaceous plants whose structural complexity provides refuge from predator attack, creating an inherent trade-off between growth and defense [14], [35], [36].

Here, we employ a novel genetic manipulation in tomato (*Solanum lycopersicum*) to test how insect herbivores respond to plants varying widely in tissue quality when simultaneously threatened with imminent attack from a key predator. We measure food intake, growth, and digestive efficiency to gain a holistic view of how consumers integrate their ‘stress phenotype’ across multiple scales and assess whether responses attenuate from behavior (i.e., foraging) to physiology (i.e., food processing). Further, we compare the outcome across an assemblage of three leaf-chewing insects (two host-plant specialists and one generalist) to gauge the consistency of patterns among species within a common guild but varying in diet breadth.

## Materials and Methods

### Study System

#### Herbivores

The focal herbivores in this study included the tobacco hornworm, *Manduca sexta* (Lepidoptera: Sphingidae), Colorado potato beetle, *Leptinotarsa decemlineata* (Coleoptera: Chrysomelidae), and cabbage looper, *Trichoplusia ni* (Lepidoptera: Noctuidae). These species are leaf-chewers during their larval stage but differ dramatically in host range. The generalist *T. ni* is highly polyphagous, feeding broadly across more than a dozen plant families [37]; in contrast, *M. sexta* and *L. decemlineata* are oligophagous herbivores that specialize on plants in the Solanaceae [38], [39]. We used second-instar larvae with *M. sexta* originating from a laboratory colony, *L. decemlineata* from a colony recently initiated using individuals collected from the field in Ithaca, NY, and *T. ni* ordered from the insectary at Benzon Research (Carlisle, Pennsylvania, USA). No permits were required for insect field collections.

#### Plants

Tomato (*Solanum lycopersicum*) is a model system in plant biology and defense against consumers with ample knowledge of underlying genetics [40], [41]. In addition to serving as an important host-plant for the aforementioned herbivores, this species offers an array of transgenic and mutant lines that vary widely in defense signaling in a standardized genetic background. We worked with a mutant variety (*jai-1*, abbreviated for ‘jasmonic acid insensitive’) that is deficient in the jasmonate hormonal cascade [42]–[44]. Jasmonates regulate a diverse suit of anti-herbivore resistance traits that are particularly effective against chewing insects [45]–[47]. We also worked with a transgenic line (*35S::Prosystemin*) that overexpresses jasmonic acid in the absence of herbivory, leading to constitutively elevated defense [43], [48]. For both mutant and transgenic, the same wild-type (cv Castlemart) was used. These three plant genotypes differ in trichomes and defensive proteins [49], and also vary in their resistance to herbivores such as *T. ni* [50] and *M. sexta* [49]. In all cases, expression of defense traits and herbivore resistance were in the expected order: *jai-1*< Castlemart < *prosystemin*.

#### Predator

**Figure 1:**
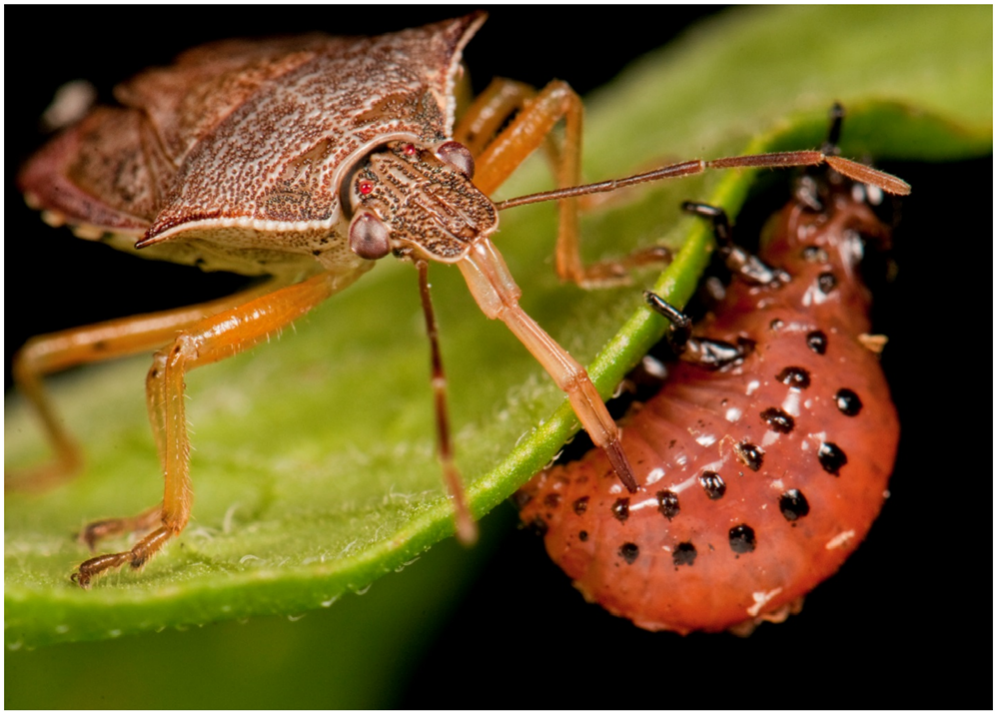
The predaceous stink bug, *P. maculiventris*, impaling a Colorado potato beetle larva, *L. decemlineata*, with its piercing-sucking stylet. These actively-foraging predators are voracious consumers of caterpillars and beetle larvae, employing extra-oral digestion to ingest the liquefied internal contents of their prey. Photo by Ellen Woods.

The stink bug *Podisus maculiventris* (Hemiptera: Pentatomidae) is a generalist predator native to the eastern U.S. that is a voracious consumer of herbivores, primarily caterpillars and beetle larvae (Fig. 1). Our prior work with this species revealed that the presence of a single adult *P. maculiventris* in experimental greenhouse and field mesocosms triggers non-lethal effects on prey (*M. sexta*) foraging behavior, marked by a 25–40% reduction in leaf tissue damage [49], [51]–[53]. This effect is likely caused by some persistent predator-emitted kairomone rather than by repeated harassment from failed attacks (I. Kaplan & J.S. Thaler, *unpublished data*). We used pheromone traps to collect *P. maculiventris* near Ithaca, New York (no permits required) and maintained a breeding population in the laboratory on bean plants and mealworms. Newly molted adults from this colony were starved for several days prior to use in experiments.

### Experimental Design

We employed a factorial design experiment that crossed plant quality, using the three tomato genotypes described above, with the presence or absence of stink bug-induced predation risk ( = 6 treatment combinations). This design was tested against all herbivores – *M. sexta*, *L*. *decemlineata*, and *T*. *ni* – resulting in three independently run trials (n = 17–21, 11–13, and 10–13 replicates per treatment combination, respectively, for each of the three species).

Tomato seedlings were germinated in 12 cm pots in an environmentally controlled growth chamber (25°C, 16∶8 LD) for four weeks, after which plants were moved to a greenhouse maintained under similar conditions. Plants were fertilized weekly using soluble NPK and selected for experiments at the 4 or 5 week stage (ca. 4 true leaves). Each individual plant served as the replicated experimental unit. To maintain herbivores and predators in their assigned treatment, all plants were individually enclosed in fine mesh netting (35×25 cm LW) that was affixed to the pot with a rubber band and knotted at the top to prevent insects from escaping.

At the start of each experiment, a single larval herbivore was weighed to the nearest 0.1 mg and then placed on a plant with a fine-tipped paint brush. At the same time, stink bugs were introduced to cages randomly assigned the predation risk treatment (one adult per cage). Previous work with this system found that surgically removing the terminal segment from the stink bug’s piercing-sucking mouthparts (the darker section, visible in Fig. 1) prevents prey-feeding but has minimal impact on predator survival or hunting behavior [49], [51]–[53]. These ‘risk’ stink bugs were used to generate predation risk cues without killing prey.

After 48 hours of exposure to plant/predator treatments, we recovered and reweighed herbivores. Replicates in which either the herbivore or predator died were removed from the analysis; however, this represented <10% of cases and thus did not introduce a major source of error. We also removed all leaves from each plant and quantified herbivory using a square grid printed over transparent plastic to estimate leaf area removal (i.e., summed total of 1 mm^2^ holes). We also collected herbivore frass using an inverted Petri dish placed directly beneath the caged plant; herbivore feces were easily distinguishable from those of the predator. The frass was dried, weighed, and analyzed for nitrogen content using a CHN elemental analyzer (Cornell University Stable Isotope Laboratory). Leaf N content did not differ across plant-types (*jai-1* = 2.00%, Castlemart = 2.00%, *prosystemin* = 2.01%). Last, we dried the herbivores in an oven at 60°C for three days and analyzed whole body composition for glycogen, sugars, and lipids following standard methods [54]. Glycogen and other sugars, including *D*-glucose standards, were determined using a hot anthrone-based assay, whereas lipid levels were measured in samples and standards with a vanillin reagent assay. Optical densities were measured with a spectrophotometer (Thermo Multiskan Spectrum) at 625 nm for glycogen and other sugars and 525 nm for lipids.

While herbivore growth and leaf consumption were measured in all three experiments, frass collection/analysis and body composition assays were restricted to a subset of the species tested. Specifically, frass was collected and weighed for *M. sexta* and *T. ni* trials but not *L. decemlineata*. Similarly, frass and body composition analyses were only conducted on *M. sexta*, but not the other two herbivores. These additional analyses were preferentially conducted on *M. sexta* because initial analyses indicated that this species was unique in its ability to compensate for predator-induced food limitation with increased efficiency (see Results).

Estimates of growth, consumption, and excretion were used to calculate herbivore food utilization and performance using Waldbauer’s nutritional indices [55]–[57], as follows:

*Relative consumption rate (RCR)*  =  leaf area consumed accounting for variation in initial mass (used as a covariate in statistical analysis, see below).

*Relative growth rate (RGR)*  =  final mass accounting for variation in initial mass (used as a covariate in statistical analysis, see below).

*Efficiency of conversion of ingested food (ECI)*  =  *weight gain*÷*leaf consumption*

*Efficiency of conversion of digested food (ECD)*  =  *weight gain*÷(*leaf consumption*−*frass*) *Approximate digestibility (AD)*  =  (*leaf consumption*−*frass*)÷*leaf consumption*

For ECI and AD, leaf consumption was converted from area to dry mass to ensure this variable was in the same units and thus directly comparable with frass. To do so, we excised leaf discs varying widely in size, obtained their dry weight, and used the regression between these two variables to calculate dry weight leaf tissue consumption (n = 122 discs; *P*<0.0001, R^2^ = 0.81).

### Statistical Analyses

We used a two-way ANOVA to test the main and interactive effects of predation risk and plant type on the following herbivore response variables: leaf consumption, final weight, ECI, ECD, AD, % frass nitrogen, and body composition (lipids, sugars, glycogen). For leaf consumption and final weight, we included initial weight as a covariate in the model. We also included leaf damage as a covariate in the % nitrogen analysis. Data were square-root transformed prior to analysis, except % nitrogen for which we used an arcsin(sqrt) transformation on the proportion. Analyses were conducted using SAS, Version 9.2 (SAS Institute, Inc., Cary, NC).

## Results

Statistical outcomes for the effects of plant type, predation risk, and their interaction on all response variables for each of the three herbivore species tested are summarized in Table 1.

**Table 1:** The main and interactive effects of plant type (‘jasmonate insensitive’, ‘wild-type’, and ‘jasmonate overexpress’ tomato) and predation risk (presence/absence of predaceous stink bug) on consumption, growth, and digestive efficiency of the (A) cabbage looper, *T. ni*, (B) Colorado potato beetle, *L. decemlineata*, and (C) tobacco hornworm, *M. sexta*.

A. *T. ni*	Plant Type (PT)	Predation Risk (PR)	PT × PR
Leaf damage	**F_2,61_ = 7.72, P = 0.0010**	**F_1,61_ = 4.37, P = 0.0408**	F_2,61_ = 0.63, P = 0.5351
Final weight	**F_2,61_ = 9.30, P = 0.0003**	F_1,61_ = 0.54, P = 0.4666	F_2,61_ = 0.41, P = 0.6667
ECI	F_2,62_ = 2.15, P = 0.1246	F_1,62_ = 1.40, P = 0.2416	F_2,62_ = 0.08, P = 0.9210
ECD	F_2,60_ = 0.17, P = 0.8442	F_1,60_ = 0.56, P = 0.4555	F_2,60_ = 0.93, P = 0.3999
AD	F_2,61_ = 1.94, P = 0.1523	F_1,61_ = 0.37, P = 0.5440	F_2,61_ = 0.81, P = 0.4501
**B. ** ***L. decemlineata***			
Leaf damage	F_2,63_ = 2.03, P = 0.1399	**F_1,63_ = 9.94, P = 0.0025**	**F_2,63_ = 3.94, P = 0.0244**
Final weight	F_2,64_ = 0.36, P = 0.7007	**F_1,64_ = 5.75, P = 0.0195**	F_2,64_ = 1.45, P = 0.2433
ECI	F_2,64_ = 0.61, P = 0.5468	F_1,64_ = 0.63, P = 0.4314	F_2,64_ = 0.60, P = 0.5545
**C. ** ***M. sexta***			
Leaf damage	F_2,104_ = 0.75, P = 0.9275	**F_1,104_ = 9.83, P = 0.0022**	F_2,104_ = 2.53, P = 0.0844
Final weight	**F_2,103_ = 11.90, P<0.0001**	F_1,103_ = 1.81, P = 0.1811	F_2,103_ = 2.74, P = 0.0691
ECI	**F_2,104_ = 4.63, P = 0.0119**	**F_1,104_ = 7.42, P = 0.0076**	F_2,104_ = 0.13, P = 0.8814
ECD	**F_2,100_ = 4.42, P = 0.0145**	**F_1,100_ = 5.38, P = 0.0224**	F_2,100_ = 0.82, P = 0.4415
AD	F_2,101_ = 2.56, P = 0.0824	F_1,101_ = 3.10, P = 0.0814	F_2,101_ = 1.24, P = 0.2927
Frass nitrogen	**F_2,106_ = 5.09, P = 0.0079**	F_1,106_ = 0.44, P = 0.5065	F_2,106_ = 0.38, P = 0.6824
Body – glycogen	**F_2,94_ = 4.21, P = 0.0178**	F_1,94_ = 1.51, P = 0.2226	F_2,94_ = 0.11, P = 0.8988
Body – sugars	F_2,93_ = 1.23, P = 0.2964	F_1,93_ = 2.61, P = 0.1095	**F_2,93_ = 3.16, P = 0.0470**
Body – lipids	F_2,93_ = 2.85, P = 0.0629	**F_1,93_ = 4.45, P = 0.0377**	F_2,93_ = 0.61, P = 0.5437

Significant effects are bolded for emphasis. ECI  =  efficiency of conversion of ingested food, ECD  =  efficiency of conversion of digested food, AD  =  approximate digestibility.

### Consumption

**Figure 2:**
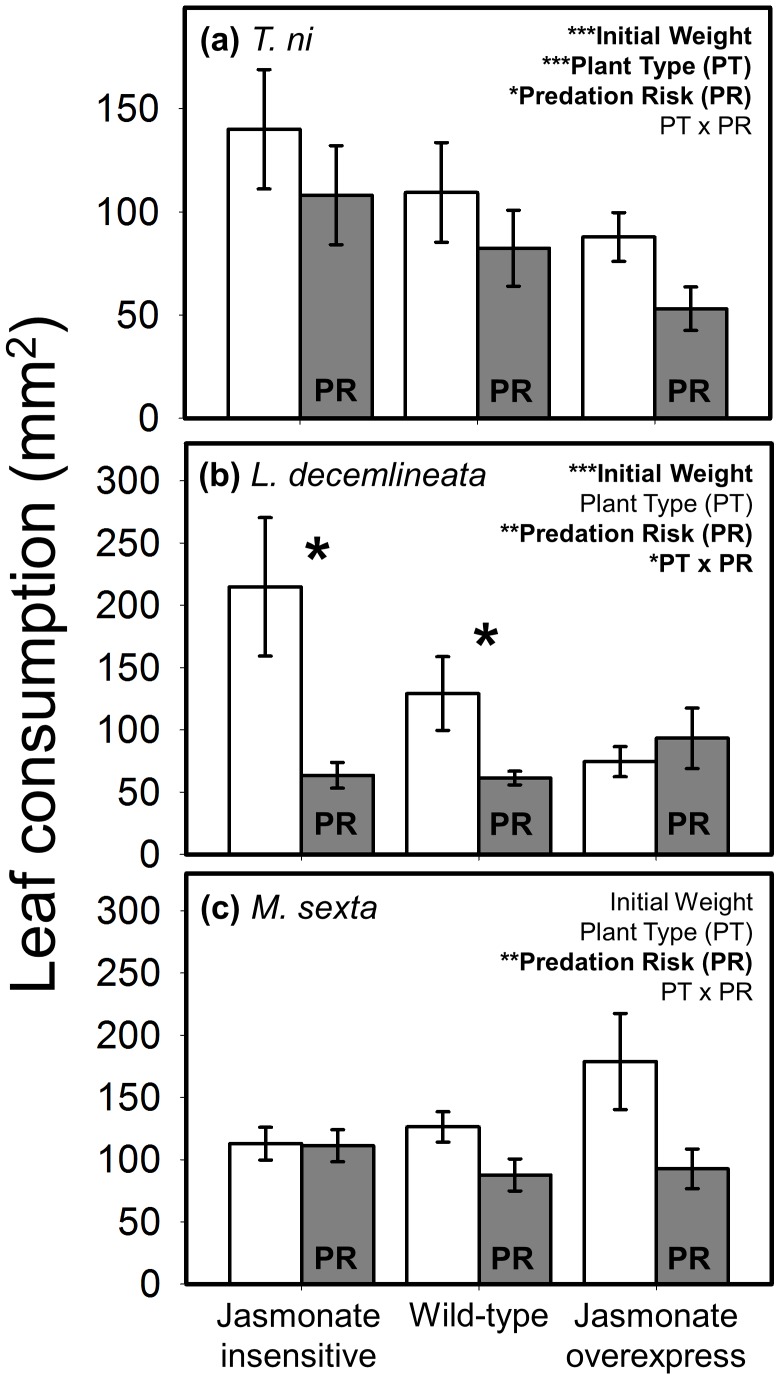
Interactive effects of plant defense and predation risk on leaf consumption (mean ± SE) of (a) *T. ni*, (b) *L. decemlineata*, and (c) *M. sexta*. Plant defense was manipulated using three plant types varying in their jasmonate signaling pathway with ‘jasmonate insensitive’ expressing low resistance, ‘wild-type’ the intermediate phenotype, and ‘jasmonate overexpress’ displaying high resistance. White bars are the predator-free control, and grey bars are labeled ‘PR’ to denote the ‘predation risk’ treatment (i.e., presence of a non-lethal stink bug). Statistical outcome for the main factors, covariate (weight), and interaction term are displayed in the upper right corner of each panel; asterisks correspond to the level of significance: * = *P*<0.05, ** = *P*<0.01, *** = *P*<0.001. Bolded asterisks above bar pairs indicate significant differences between the predator-free and predation risk treatments at each level of plant resistance in cases where the resource-risk interaction was significant. N = 10–13, 11–13, and 17–21 replicates per treatment combination for *T*. *ni*, *L*. *decemlineata*, and *M. sexta*, respectively.

The presence of predators reduced leaf consumption for all three species (compare white vs. grey bars in Fig. 2), resulting in an average of 29% less plant damage relative to predator-free controls. Plant type alone only affected the generalist, *T. ni*, with a 43% reduction in feeding on the high resistance JA-overexpressing plant line compared with the JA-insensitive mutant and intermediate levels for the wild-type (Fig. 2a). In contrast, plant type did not affect the two specialists (Figs. 2b, 2c). For the potato beetle, *L. decemlineata*, there was a statistical interaction between plant type and predation risk whereby the influence of predators became progressively weaker with increasing plant resistance. Stink bug presence reduced *L. decemlineata* feeding by 70% and 52%, respectively, on low- and intermediate-resistance plant genotypes, but did not impact consumption of the high resistance line (Fig. 2b).

### Herbivore Growth

**Figure 3:**
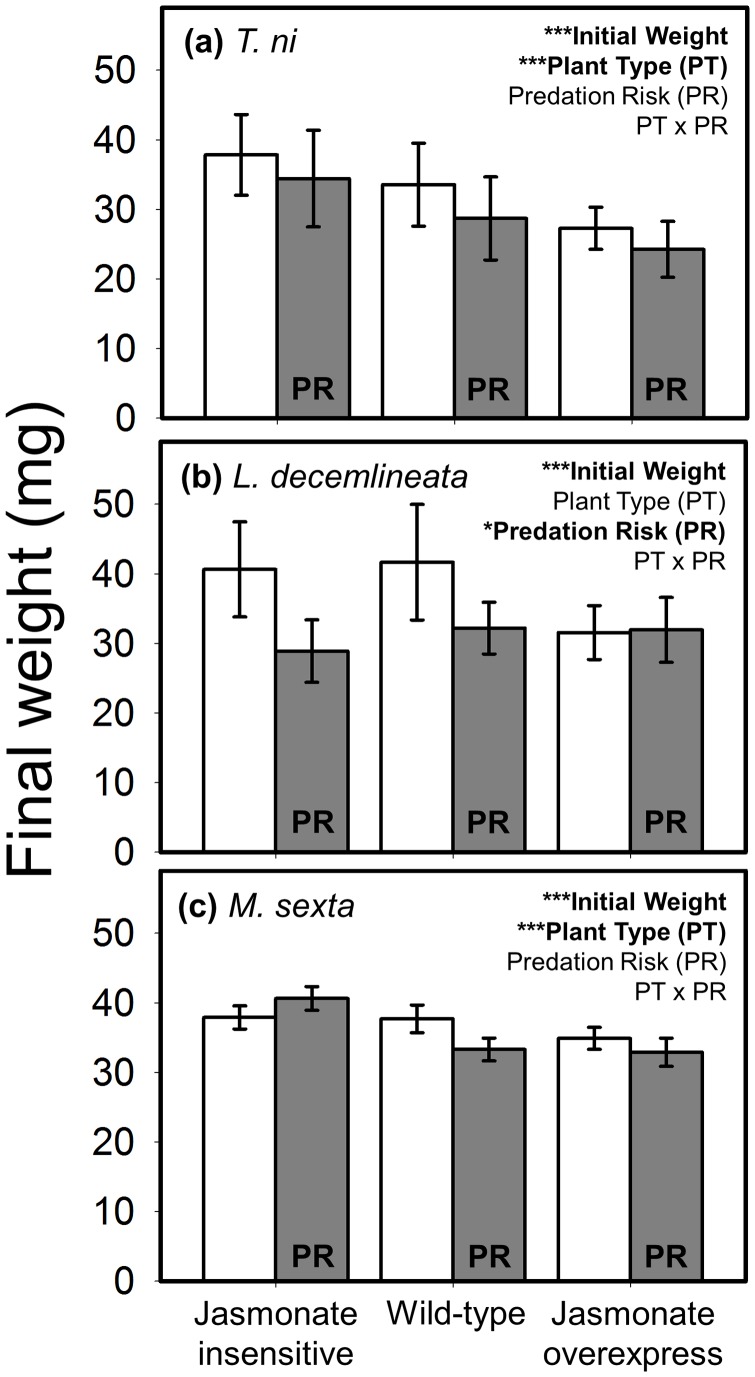
Interactive effects of plant defense and predation risk on larval weight (mean ± SE) of (a) *T. ni*, (b) *L. decemlineata*, and (c) *M. sexta*. Plant defense was manipulated using three plant types varying in their jasmonate signaling pathway with ‘jasmonate insensitive’ expressing low resistance, ‘wild-type’ the intermediate phenotype, and ‘jasmonate overexpress’ displaying high resistance. White bars are the predator-free control, and grey bars are labeled ‘PR’ to denote the ‘predation risk’ treatment (i.e., presence of a non-lethal stink bug). Statistical outcome for the main factors, covariate (weight), and interaction term are displayed in the upper right corner of each panel; asterisks correspond to the level of significance: * = *P*<0.05, ** = *P*<0.01, *** = *P*<0.001. N = 10–13, 11–13, and 17–21 replicates per treatment combination for *T*. *ni*, *L*. *decemlineata*, and *M. sexta*, respectively.

Effects of predation risk on weight gain were far less consistent than foraging behavior: of the three herbivore species, only *L. decemlineata* (Fig. 3b) experienced a significant (17%) risk-induced reduction in body weight. Plant resistance, on the other hand, affected the growth of *T. ni* (Fig. 3a) and *M. sexta* (Fig. 3c): both species grew best on JA-insensitive plants and did worst on JA-overexpressing plants. Plant genotype had a far stronger effect on the generalist than the specialist, with a 29% decline in *T. ni* weight compared to a 14% reduction for *M. sexta*. The predation risk x plant type interaction term was non-significant for all species.

### Digestive Efficiency

**Figure 4:**
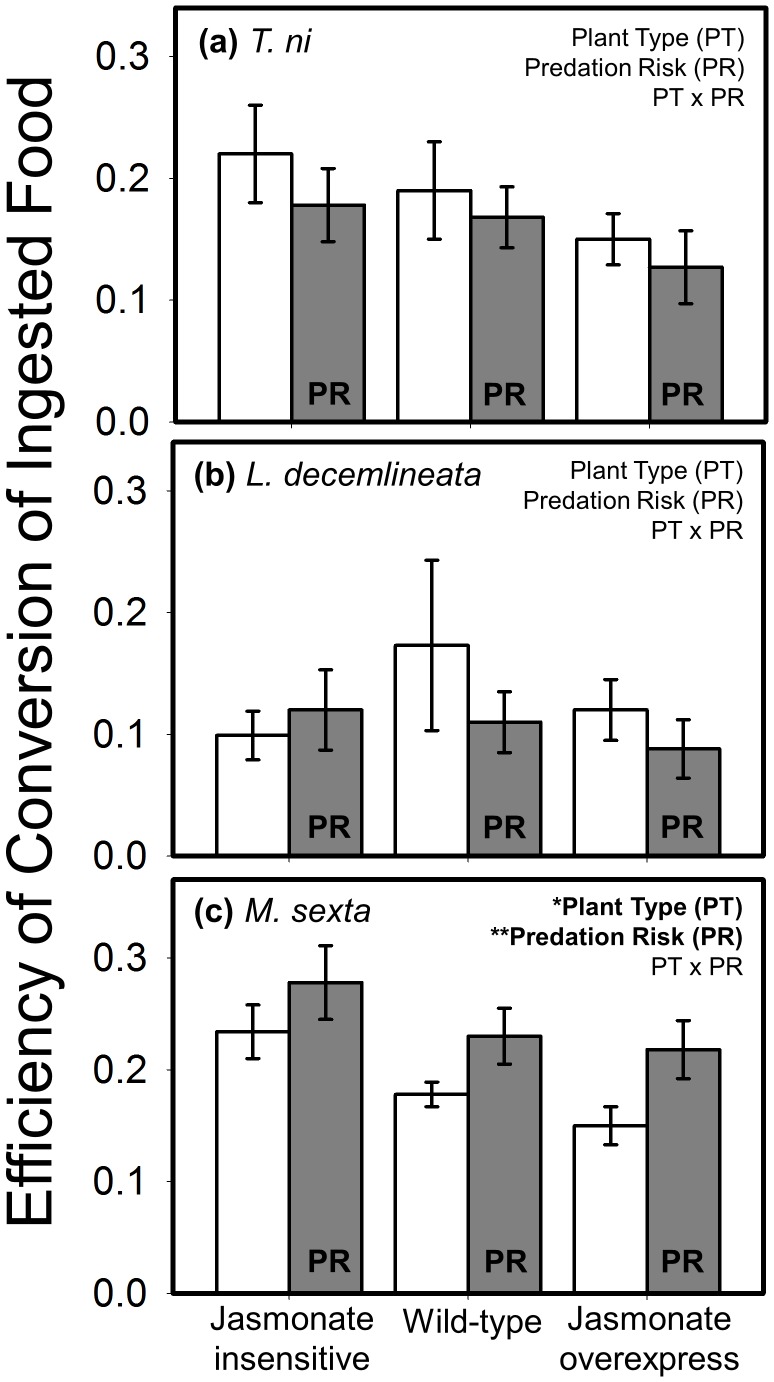
Interactive effects of plant defense and predation risk on the efficiency of conversion of ingested food (mean ± SE) for (a) *T. ni*, (b) *L. decemlineata*, and (c) *M. sexta*. Plant defense was manipulated using three plant types varying in their jasmonate signaling pathway with ‘jasmonate insensitive’ expressing low resistance, ‘wild-type’ the intermediate phenotype, and ‘jasmonate overexpress’ displaying high resistance. White bars are the predator-free control, and grey bars are labeled ‘PR’ to denote the ‘predation risk’ treatment (i.e., presence of a non-lethal stink bug). Statistical outcome for the main factors, covariate (weight), and interaction term are displayed in the upper right corner of each panel; asterisks correspond to the level of significance: * = *P*<0.05, ** = *P*<0.01, *** = *P*<0.001. N = 10–13, 11–13, and 17–21 replicates per treatment combination for *T*. *ni*, *L*. *decemlineata*, and *M. sexta*, respectively.

**Figure 5:**
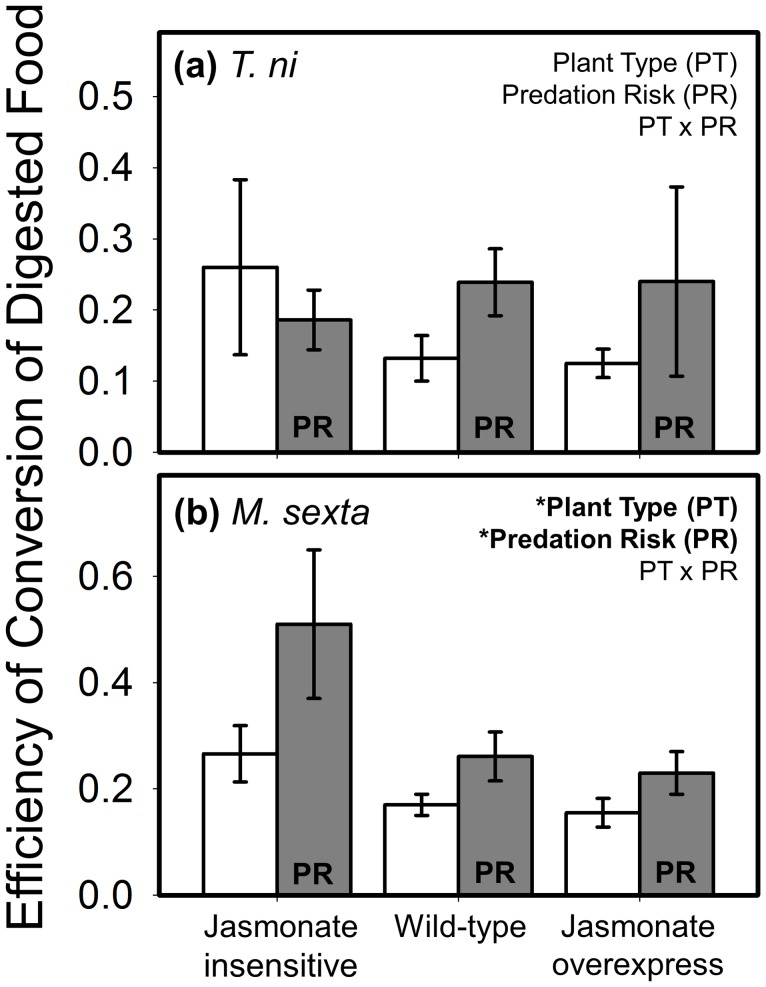
Interactive effects of plant defense and predation risk on the efficiency of conversion of digested food (mean ± SE) for (a) *T. ni*, and (b) *M. sexta*. Plant defense was manipulated using three plant types varying in their jasmonate signaling pathway with ‘jasmonate insensitive’ expressing low resistance, ‘wild-type’ the intermediate phenotype, and ‘jasmonate overexpress’ displaying high resistance. White bars are the predator-free control, and grey bars are labeled ‘PR’ to denote the ‘predation risk’ treatment (i.e., presence of a non-lethal stink bug). Statistical outcome for the main factors, covariate (weight), and interaction term are displayed in the upper right corner of each panel; asterisks correspond to the level of significance: * = *P*<0.05, ** = *P*<0.01, *** = *P*<0.001. N = 10–13 and 17–21 replicates per treatment combination for *T*. *ni* and *M. sexta*, respectively.

We used several measures to estimate digestive efficiency. The only measure comparable across the three herbivores was ECI (efficiency of conversion of ingested food): two of the species were unaffected by both the main treatment factors and their interaction (Figs. 4a, 4b). ECI in *M. sexta* was affected by both plant type and predation risk, but not their interaction (Fig. 4c). Caterpillars were more efficient when feeding on low-resistance plants, and when exposed to predation risk. An analogous outcome was found for the related measure, ECD (efficiency of conversion of digested food), which accounts for mass loss due to excretion. ECD of *T. ni* was unaffected by either treatment (Fig. 5a); *M. sexta*, however, increased its efficiency on low-resistance plants and in response to predation risk but not their interaction (Fig. 5b). Because approximate digestibility (AD) was unaffected by any treatment in any species, the data are not shown (but the statistical outcome is reported in Table 1).

**Figure 6:**
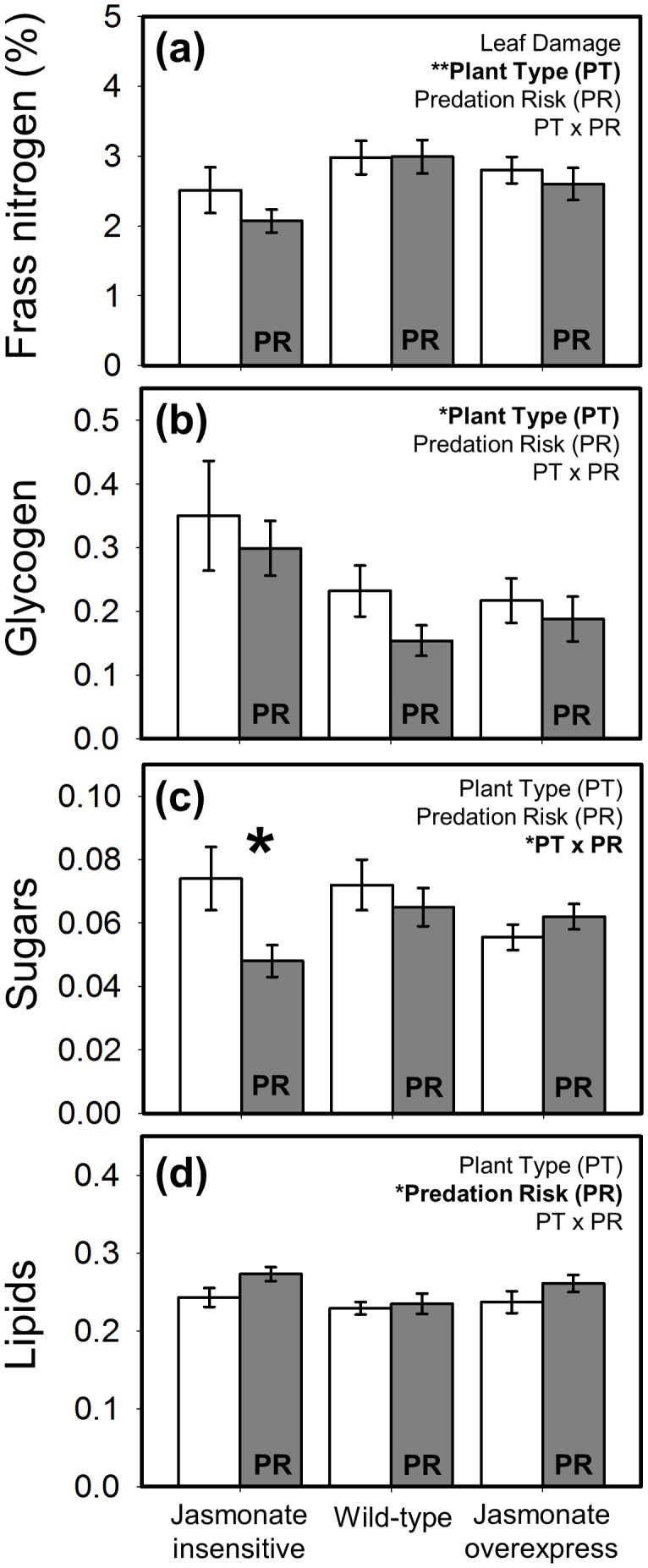
Interactive effects of plant defense and predation risk on *M. sexta* digestive physiology (mean ± SE) as measured via (a) frass nitrogen, and whole-body composition (μg/mg caterpillar dry weight) – (b) glycogen, (c) sugars, and (d) lipids. Plant defense was manipulated using three plant types varying in their jasmonate signaling pathway with ‘jasmonate insensitive’ expressing low resistance, ‘wild-type’ the intermediate phenotype, and ‘jasmonate overexpress’ displaying high resistance. White bars are the predator-free control, and grey bars are labeled ‘PR’ to denote the ‘predation risk’ treatment (i.e., presence of a non-lethal stink bug). Statistical outcome for the main factors, covariate (weight), and interaction term are displayed in the upper right corner of each panel; asterisks correspond to the level of significance: * = *P*<0.05, ** = *P*<0.01, *** = *P*<0.001. Bolded asterisks above bar pairs indicate significant differences between the predator-free and predation risk treatments at each level of plant resistance in cases where the resource-risk interaction was significant. N = 17–21 replicates per treatment combination.

Treatment effects on frass nitrogen and body composition were only available for hornworm caterpillars. Although there was no effect of predation risk, *M. sexta* excreted less nitrogen when feeding on JA-insensitive plants (Fig. 6a). Glycogen levels were similarly unaffected by predation risk but >50% higher in larvae reared on JA-insensitive plants (Fig. 6b). Sugars were impacted by a resource-risk interaction: predation risk reduced body sugars, but only on low-resistance plants (Fig. 6c). Finally, lipids increased under predation risk, but were unaffected by plant resistance and the resource-risk interaction (Fig. 6d).

## Discussion

The relative importance of plant quality vs. predation risk on herbivores has rarely been directly quantified, especially along multiple traits (e.g., foraging behavior, digestive physiology) across an assemblage of consumers. Our data suggest that activity reduction, and thus reduced food intake, is a near-universal response to predators. The main impact of predation risk on leaf consumption was found in all three species, and was the most consistent effect across multiple treatments, trials, and response variables. Although prior work with this tri-trophic system has demonstrated an analogous outcome for *M. sexta* [49], [51]–[53], this phenomenon has not been widely documented for other leaf-chewing insects in the tomato system. The consistency of this response mirrors the broader literature on vertebrate herbivores, including mammals and amphibians [12], [13], and corresponds with theoretical expectations based on the likelihood of predator encounter for prey engaged in different behavioral states. Feeding is known to be risky: herbivorous insects are estimated to be ca. 100x more likely to be killed by a predator when foraging than when resting [19], [20]. The adaptive shifts in foraging behavior documented here can thus be viewed as central components of a risk-reduction strategy that balances the competing demands of growth and defense. The fact that a generalist predator like *P. maculiventris* is capable of triggering such a consistent interspecific response suggests that prey may be particularly attuned to generalized risk cues in the environment [58].

Unlike the effects of predation risk, responses to plant quality, including both food intake and growth rate, varied across species with the magnitude correlated with herbivore diet breadth. Specialists are, on average, more tolerant of plant defensive chemistry than generalists [59], [60]. In keeping with this pattern, JA-mediated defenses reduced feeding and growth in the generalist, *T. ni*, but affected neither response in the Solanaceae-specialist *L. decemlineata*. Similarly, plant type only impacted weight gain (but not consumption) in the specialist *M. sexta* with the strength of this effect considerably less pronounced than with *T. ni*. Because we lack species-level replication for generalist herbivores, the link between diet breadth and defense tolerance is somewhat tenuous; however, in related work rearing another generalist caterpillar, *Spodoptera exigua*, on the same plant lines we documented comparably strong effects on larval growth and feeding habits (I. Kaplan & J.S. Thaler, *unpublished data*).

Changes in herbivore digestive physiology were only apparent in *M. sexta* (see Fig. 4) where the efficiency by which larvae convert plant food to body mass was highest on low-resistance plants and in response to predation risk. The impact of plant resistance may be driven by the fact that concentrations of anti-nutritive defensive proteins (e.g., protease inhibitors, polyphenol oxidase), are much lower in the jasmonate-insensitive genotype than the other two lines [49]. These data, along with those showing that frass from caterpillars eating low-resistance plants contains less nitrogen (Fig. 5a), confirms that JA-mediated defenses function, at least in part, by reducing the nutritive content of plant tissue for consumers. The frass outcome, in particular, mechanistically links this phytohormonal pathway with the insect’s capacity to extract bound nitrogen in their digestive tract. Further, the substantially (>50%) higher glycogen levels in caterpillars developing on low-resistance plants demonstrate that effects are not limited to nitrogen metabolism, but also have important physiological consequences for energetic reserves [61].

Predators induced analogous increases in hornworm efficiency compared with the above-described pattern observed for high quality plants. Because predation risk decreases prey movement, enhanced efficiency may simply result from lower energy expenditure in risky environments [62]. Recent work in the hornworm-stink bug system, however, demonstrates that such changes in conversion efficiency are predator-specific: they cannot be recreated by simulating predator-induced feeding reductions by experimentally withholding food in the absence of predators [53]. This work also found that compensation only occurred when insects were exposed to brief bouts of predation risk (three days or less). Caterpillars were unable to compensate over longer time scales, illustrating that growth maintenance in the face of predation risk is a temporary solution to an ephemeral threat. Future studies would benefit from following prey development into adulthood to assess if compensation early in life has costs that are manifested in later growth stages.

Despite the increased efficiency of body mass conversion induced by predators, this did not translate to an obvious physiological mechanism. While predation risk tended to decrease fecal nitrogen, this overall effect was non-significant. We did, however, detect a consistent increase in lipids for caterpillars reared under predation risk. Because lipids store energy for use during extended non-feeding periods [63], we speculate that foragers convert a greater proportion of food energy to lipids during risk-induced periods of low activity. Although few studies have empirically linked ecological mechanisms with the nutritional body composition of invertebrates, Mediterranean fruit flies, *Ceratitis capitata*, also displayed higher lipid levels while resting between active feeding bouts [64].

We were surprised by how few statistically significant resource-risk interactions appeared in the dataset. The primary interaction we detected, in which the impact of predation risk on beetle foraging attenuated with increasing plant resistance (Fig. 2b), is consistent with the results of an earlier field experiment on *M. sexta* foraging using the same tomato lines and stink bugs [49]. While this study employed a larger multiple-plant arena in which predators could be deterred by plant trichomes and other features, the present study utilized single bagged plants on which predation risk was presumably more omnipresent. These convergent outcomes may partially be driven by the fact that the predator used in these studies is omnivorous, routinely consuming both plant and prey foods. Thus, plant resistance phenotypes likely have direct effects on the predator. Indeed, in a separate study we have documented direct negative impacts of the jasmonate phenotype on *P. maculiventris* development (J.S. Thaler, E. Olsen & I. Kaplan, *unpublished manuscript*). While the present study also found that exposure to predation risk increased larval efficiency regardless of plant resistance, other studies in this system have found similar increases in assimilation efficiency only in larvae feeding on high-quality plants [65]. These disparate results suggest that the effects of predation risk and plant resistance on *M. sexta* digestive efficiency may be context-dependent and vary with factors such as spatial scale, predator cue duration, and other environmental conditions.

Overall, our work across three different herbivore species shows a consistent reduction in food intake in response to predation risk. At the same time, we show how species-specific responses to variation in plant quality and, to a lesser extent, interactions between plant quality and predation risk, affect herbivore compensation for reduced food intake. These results highlight: 1) the dual importance of food quality and predation risk for herbivores in tri-trophic systems, and 2) the need for a broader understanding of compensatory mechanisms to more accurately predict how predation risk will affect communities and ecosystems. Recent work has emphasized moving beyond mere foraging behavior to track changes in prey physiology (e.g., metabolic rate, development time) under the threat of predators [21]–[25], [53]. Such an integrative approach could confirm, for example, whether the patterns we found for generalist vs. specialist herbivores represent a more universal phenomenon. We suggest that a broader consideration of compensatory mechanisms could be especially useful in terrestrial invertebrate communities, where the ecological and evolutionary significance of predators is well-recognized, but the integrated phenotypic response in prey has yet to be fully uncovered.

## References

[1] Relyea RA, Mills N (2001) Predator-induced stress makes the pesticide carbaryl more deadly to gray treefrog tadpoles (*Hyla versicolor*). Proceedings of the National Academy of Sciences (USA) 98: 2491–2496. 10.1073/pnas.031076198 PMC30165 11226266

[2] Relyea RA (2003) Predator cues and pesticides: A double dose of danger for amphibians. Ecological Applications 13: 1515–1521.

[3] Relyea RA (2004) Synergistic impacts of malathion and predatory stress on six species of North American tadpoles. Environmental Toxicology and Chemistry 23: 1080–1084. 15095908 10.1897/03-259

[4] Coors A, De Meester L (2008) Synergistic, antagonistic and additive effects of multiple stressors: predation threat, parasitism and pesticide exposure in *Daphnia magna* . Journal of Applied Ecology 45: 1820–1828.

[5] Darling ES, Cote IM (2008) Quantifying the evidence for ecological synergies. Ecology Letters 11: 1278–1286. 18785986 10.1111/j.1461-0248.2008.01243.x

[6] Stamp NE, Casey TM (1993) Caterpillars: Ecological and Evolutionary Constraints on Foraging. New York: Chapman & Hall. 587 p.

[7] Tilmon KJ (2008) The Evolutionary Biology of Herbivorous Insects: Specialization, Speciation, and Radiation. Berkeley: University of California Press. 341 p.

[8] Denno RF, McClure MS (1983) Variable plants and herbivores in natural and managed systems. New York: Academic Press. 717 p.

[9] Fritz RS, Simms EL (1992) Plant resistance to herbivores and pathogens: Ecology, evolution, and genetics. Chicago: University of Chicago Press. 590 p.

[10] White TCR (1993) The inadequate environment: Nitrogen and the abundance of animals. Berlin: Springer-Verlag. 425 p.

[11] Karban R, Baldwin IT (1997) Induced responses to herbivory. Chicago: University of Chicago Press. 330 p.

[12] Lima SL, Dill LM (1990) Behavioral decisions made under the risk of predation – a review and prospectus. Canadian Journal of Zoology 68: 619–640.

[13] Preisser EL, Bolnick DI, Benard MF (2005) Scared to death? The effects of intimidation and consumption in predator-prey interactions. Ecology 86: 501–509.

[14] Schmitz OJ (1998) Direct and indirect effects of predation and predation risk in old-field interaction webs. American Naturalist 151: 327–342. 10.1086/286122 18811324

[15] Relyea RA, Werner EE (1999) Quantifying the relation between predator-induced behavior and growth performance in larval anurans. Ecology 80: 2117–2124.

[16] Sheriff MJ, Krebs CJ, Boonstra R (2009) The sensitive hare: sublethal effects of predator stress on reproduction in snowshoe hares. Journal of Animal Ecology 78: 1249–1258. 19426257 10.1111/j.1365-2656.2009.01552.x

[17] Christianson D, Creel S (2010) A nutritionally mediated risk effect of wolves on elk. Ecology 91: 1184–1191. 20462132 10.1890/09-0221.1

[18] Zanette LY, White AF, Allen MC, Clinchy M (2011) Perceived predation risk reduces the number of offspring songbirds produce per year. Science 334: 1398–1401. 22158817 10.1126/science.1210908

[19] Bernays EA (1997) Feeding by lepidopteran larvae is dangerous. Ecological Entomology 22: 121–123.

[20] Gotthard K (2000) Increased risk of predation as a cost of high growth rate: an experimental test in a butterfly. Journal of Animal Ecology 69: 896–902. 29313992 10.1046/j.1365-2656.2000.00432.x

[21] Preisser EL (2009) The physiology of predator stress in free-ranging prey. Journal of Animal Ecology 78: 1103–1105. 19840173 10.1111/j.1365-2656.2009.01602.x

[22] Hawlena D, Schmitz OJ (2010) Physiological stress as a fundamental mechanism linking predation to ecosystem functioning. American Naturalist 176: 537–556. 10.1086/656495 20846014

[23] Hawlena D, Schmitz OJ (2010) Herbivore physiological response to fear of predation alters ecosystem nutrient dynamics. Proceedings of the National Academy of Sciences (USA) 107: 15503–15507. 10.1073/pnas.1009300107 PMC2932623 20713698

[24] Slos S, Stoks R (2008) Predation risk induces stress proteins and reduces antioxidant defense. Functional Ecology 22: 637–642.

[25] Janssens L, Stoks R (2013) Predation risk causes oxidative damage in prey. Biology Letters 9: 20130350. 23760170 10.1098/rsbl.2013.0350 PMC3730648

[26] Anholt BR, Werner EE (1998) Predictable changes in predation mortality as a consequence of changes in food availability and predation risk. Evolutionary Ecology 12: 729–738.

[27] Danner BJ, Joern A (2003) Resource-mediated impact of spider predation risk on performance in the grasshopper *Ageneotettix deorum* (Orthoptera: Acrididae). Oecologia 137: 352–359. 12928859 10.1007/s00442-003-1362-9

[28] Steiner UK (2007) Linking antipredator behaviour, ingestion, gut evacuation and costs of predator-induced responses in tadpoles. Animal Behaviour 74: 1473–1479.

[29] Brönmark C, Lakowitz T, Nilsson PA, Ahlgren J, Lennartsdotter C, et al (2012) Costs of inducible defence along a resource gradient. PLoS ONE 7: e30467. 22291961 10.1371/journal.pone.0030467 PMC3265497

[30] Slansky F, Feeny P (1977) Stabilization of the rate of nitrogen accumulation by larvae of the cabbage butterfly on wild and cultivated food plants. Ecological Monographs 47: 209–228.

[31] Moran N, Hamilton WD (1980) Low nutritive quality as defense against herbivores. Journal of Theoretical Biology 86: 247–254.

[32] Lavoie B, Oberhauser KS (2004) Compensatory feeding in *Danaus plexippus* (Lepidoptera: Nymphalidae) in response to variation in host plant quality. Environmental Entomology 33: 1062–1069.

[33] Steppuhn A, Baldwin IT (2007) Resistance management in a native plant: nicotine prevents herbivores from compensating for plant protease inhibitors. Ecology Letters 10: 499–511. 17498149 10.1111/j.1461-0248.2007.01045.x

[34] Thaler JS, Farag MA, Paré PW, Dicke M (2002) Jasmonate-deficient plants have reduced direct and indirect defences against herbivores. Ecology Letters 5: 764–774.

[35] Beckerman AP, Uriarte M, Schmitz OJ (1997) Experimental evidence for a behavior-mediated trophic cascade in a terrestrial food chain. Proceedings of the National Academy of Sciences (USA) 94: 10735–10738. 10.1073/pnas.94.20.10735 PMC23467 11038581

[36] Schmitz OJ, Suttle KB (2001) Effects of top predator species on direct and indirect interactions in a food web. Ecology 82: 2072–2081.

[37] Soo Hoo CR, Coudriet DL, Vail PV (1984) *Trichoplusia ni* (Lepidoptera: Noctuidae) larval development on wild and cultivated plants. Environmental Entomology 13: 843–846.

[38] Yamamoto RT, Fraenkel GS (1960) The specificity of the tobacco hornworm, *Protoparce sexta*, to solanaceous plants. Annals of the Entomological Society of America 53: 503–507.

[39] Hsiao TH, Fraenkel G (1968) The role of secondary plant substances in the food specificity of the Colorado potato beetle. Annals of the Entomological Society of America 61: 485–493.

[40] Kennedy GG (2003) Tomato, pests, parasitoids, and predators: Tritrophic interactions involving the genus *Lycopersicon* . Annual Review of Entomology 48: 51–72. 10.1146/annurev.ento.48.091801.112733 12194909

[41] The Tomato Genome Consortium (2012) The tomato genome sequence provides insights into fleshy fruit evolution. Nature 485: 635–641. 22660326 10.1038/nature11119 PMC3378239

[42] Howe GA, Lightner J, Browse J, Ryan CA (1996) An octadecanoid pathway mutant (JL5) of tomato is compromised in signaling for defense against insect attack. Plant Cell 8: 2067–2077. 8953771 10.1105/tpc.8.11.2067 PMC161335

[43] Li L, Li CY, Lee GI, Howe GA (2002) Distinct roles for jasmonate synthesis and action in the systemic wound response of tomato. Proceedings of the National Academy of Sciences (USA) 99: 6416–6421. 10.1073/pnas.072072599 PMC122963 11959903

[44] Li L, Zhao YF, McCaig BC, Wingerd BA, Wang JH, et al (2004) The tomato homolog of CORONATINE-INSENSITIVE1 is required for the maternal control of seed maturation, jasmonate-signaled defense responses, and glandular trichome development. Plant Cell 16: 126–143. 14688297 10.1105/tpc.017954 PMC301400

[45] McConn M, Creelman RA, Bell E, Mullet JE, Browse J (1997) Jasmonate is essential for insect defense in *Arabidopsis* . Proceedings of the National Academy of Sciences (USA) 94: 5473–5477. 10.1073/pnas.94.10.5473 PMC24703 11038546

[46] Thaler JS, Stout MJ, Karban R, Duffey SS (2001) Jasmonate-mediated induced plant resistance affects a community of herbivores. Ecological Entomology 26: 312–324.

[47] Erb M, Meldau S, Howe GA (2012) Role of phytohormones in insect-specific plant reactions. Trends in Plant Science 17: 250–259. 22305233 10.1016/j.tplants.2012.01.003 PMC3346861

[48] Orozco-Cardenas M, McGurl B, Ryan CA (1993) Expression of an antisense prosystemin gene in tomato plants reduces resistance toward *Manduca sexta* larvae. Proceedings of the National Academy of Sciences (USA) 90: 8273–8276. 10.1073/pnas.90.17.8273 PMC47331 11607423

[49] Kaplan I, Thaler JS (2010) Plant resistance attenuates the consumptive and non-consumptive impacts of predators on prey. Oikos 119: 1105–1113.

[50] Scott IM, Thaler JS, Scott JG (2010) Response of a generalist herbivore *Trichoplusia ni* to jasmonate-mediated induced defense in tomato. Journal of Chemical Ecology 36: 490–499. 20422268 10.1007/s10886-010-9780-8

[51] Griffin CAM, Thaler JS (2006) Insect predators affect plant resistance via density- and trait-mediated indirect interactions. Ecology Letters 9: 338–346. 16958900 10.1111/j.1461-0248.2005.00880.x

[52] Thaler JS, Griffin CAM (2008) Relative importance of consumptive and non-consumptive effects of predators on prey and plant damage: the influence of herbivore ontogeny. Entomologia Experimentalis et Applicata 128: 34–40.

[53] Thaler JS, McArt SH, Kaplan I (2012) Compensatory mechanisms for ameliorating the fundamental trade-off between predator avoidance and foraging. Proceedings of the National Academy of Sciences (USA) 109: 12075–12080. 10.1073/pnas.1208070109 PMC3409718 22778426

[54] van Handel E, Day JF (1988) Assay of lipids, glycogen and sugars in individual mosquitoes: correlations with wing length in field-collected *Aedes vexans* . Journal of the American Mosquito Control Association 4: 549–550. 3225576

[55] Waldbauer GP (1968) The consumption and utilization of food by insects. Advances in Insect Physiology 5: 229–288.

[56] Slansky F, Scriber JM (1985) Food consumption and utilization. In: Kerkut GA, Gilbert LI, editors. Comprehensive insect physiology biochemistry & pharmacology. Volume 4. Regulation: digestion, nutrition, excretion. New York: Pergamon. 88–151.

[57] Rayapuram C, Baldwin IT (2006) Using nutritional indices to study LOX-3 dependent insect resistance. Plant Cell & Environment 29: 1585–1594. 10.1111/j.1365-3040.2006.01534.x 16898019

[58] Castellanos I, Barbosa P (2006) Evaluation of predation risk by a caterpillar using substrate-borne vibrations. Animal Behaviour 72: 461–469.

[59] Cornell H, Hawkins B (2003) Herbivore responses to plant secondary compounds: a test of phytochemical coevolution theory. American Naturalist 161: 507–522. 10.1086/368346 12776881

[60] Ali JG, Agrawal AA (2012) Specialist versus generalist insect herbivores and plant defense. Trends in Plant Science 17: 293–302. 22425020 10.1016/j.tplants.2012.02.006

[61] Chapman RF, Simpson SJ, Douglas AE (2012) The insects: Structure and function, 5th Edition. Cambridge: Cambridge University Press.

[62] Johansson F, Andersson J (2009) Scared fish get lazy, and lazy fish get fat. Journal of Animal Ecology 78: 772–777. 19302323 10.1111/j.1365-2656.2009.01530.x

[63] Arrese EL, Soulages JL (2010) Insect fat body: Energy, metabolism, and regulation. Annual Review of Entomology 55: 207–225. 10.1146/annurev-ento-112408-085356 PMC3075550 19725772

[64] Warburg MS, Yuval B (1997) Effects of energetic reserves on behavioral patterns of Mediterranean fruit flies (Diptera: Tephritidae). Oecologia 112: 314–319. 28307478 10.1007/s004420050314

[65] Thaler JS, Contreras H, Davidowitz G (In press) Effects of predation risk and plant resistance on *Manduca sexta* caterpillar feeding behavior and physiology. Ecological Entomology.

